# Supplementary material for: Human neuronal firing varies with the frequency of local field potential oscillations
Source: PLoS Biol. 2026 Jun 23;24(6):e3003818. doi: 10.1371/journal.pbio.3003818 (PMC13289887; doi:10.1371/journal.pbio.3003818)
Supplement: S1 Table — (DOCX) [file pbio.3003818.s001.docx]

**S1 Table.** Patients’ demographics

| **Gender** | | **Race** | | **Age (years)** | |
| --- | --- | --- | --- | --- | --- |
| Female | 9 | White | 11 | 20-30 | 7 |
| Male | 10 | Black | 3 | 31-40 | 3 |
|  |  | Hispanic | 2 | 41-50 | 6 |
|  |  | Asian | 2 | 51-60 | 3 |
|  |  | Multi-race | 1 |  |  |
