# Supplementary material for: Human neuronal firing varies with the frequency of local field potential oscillations
Source: PLoS Biol. 2026 Jun 23;24(6):e3003818. doi: 10.1371/journal.pbio.3003818 (PMC13289887; doi:10.1371/journal.pbio.3003818)
Supplement: S2 Table — The table summarizes the number of neurons exhibiting significant frequency tuning, phase tuning, or joint phase-and-frequency tuning for each subject. Counts are reported separately for neurons recorded from macroelectrodes and single units. This breakdown illustrates the contribution of individual subjects to the overall pool of tuned neurons reported in the study and demonstrates that the observed effects are not driven by a single subject. (DOCX) [file pbio.3003818.s002.docx]

**S2 Table.** Distribution of frequency- and phase-tuned neurons across subjects.

| **Subject** | **Frequency tuned** | | **Phase tuned** | | **Phase & frequency tuned** | |
| --- | --- | --- | --- | --- | --- | --- |
|  | **Macro** | **Unit** | **Macro** | **Unit** | **Macro** | **Unit** |
| **1** | 1 | 2 | 2 | 3 | 1 | 1 |
| **2** | 2 | 3 | 2 | 3 | - | - |
| **3** | 1 | 1 | 3 | 4 | - | - |
| **4** | 1 | 1 | 1 | 1 | - | - |
| **5** | - | - | 3 | 5 | - | - |
| **6** | 2 | 3 | 3 | 8 | - | - |
| **7** | 2 | 5 | 3 | 4 | - | - |
| **8** | 1 | 1 | 3 | 7 | - | - |
| **9** | - | - | 1 | 1 | - | - |
| **10** | 2 | 5 | - | - | 2 | 2 |
| **11** | 1 | 1 | 1 | 1 | - | - |
| **12** | 2 | 2 | 2 | 2 | - | - |
| **13** | 1 | 1 | - | - |  |  |
| **14** | - | - | 1 | 1 | - | - |
| **15** | - | - | 1 | 1 | - | - |
| **16** | - | - | 1 | 1 | - | - |
| **17** | 1 | 1 | 2 | 2 | - | - |
| **18** | 1 | 1 | 5 | 10 | - | - |

The table summarizes the number of neurons exhibiting significant frequency tuning, phase tuning, or joint phase-and-frequency tuning for each subject. Counts are reported separately for neurons recorded from macroelectrodes and single units. This breakdown illustrates the contribution of individual subjects to the overall pool of tuned neurons reported in the study and demonstrates that the observed effects are not driven by a single subject.
