# Supplementary material for: Human neuronal firing varies with the frequency of local field potential oscillations
Source: PLoS Biol. 2026 Jun 23;24(6):e3003818. doi: 10.1371/journal.pbio.3003818 (PMC13289887; doi:10.1371/journal.pbio.3003818)
Supplement: S3 Table — The table shows the numeric values comparing the dominant LFP frequency and the frequency-tuning value for each neuron, represented visually in Fig 6A. The figure and table display units with increased firing rate at a specific frequency (n = 22/27). (DOCX) [file pbio.3003818.s003.docx]

**S3 Table.**

| **Neuron** | **Dominant LFP frequency (Hz) – Frequency at max R^2^** | **Preferred tuning frequency (Hz)**  **- Frequency at increased spiking** |
| --- | --- | --- |
| **1** | 7.8 Hz | 9.2 Hz |
| **2** | 7.8 Hz | 8.8 Hz |
| **3** | 1.7 Hz | 2.1 Hz |
| **4** | 2.4 Hz | 3.6 Hz |
| **5** | 2.8 Hz | 3.4 Hz |
| **6** | 4.2 Hz | 2.4 Hz |
| **7** | 4.2 Hz | 4.6 Hz |
| **8** | 4.2 Hz | 4.9 Hz |
| **9** | 2.9 Hz | 2.5 Hz |
| **10** | 8.4 Hz | 10.4 Hz |
| **11** | 3.7 Hz | 1.8 Hz |
| **12** | 1.7 Hz | 10.1 Hz |
| **13** | 3.4 Hz | 4.9 Hz |
| **14** | 3.4 Hz | 3.6 Hz |
| **15** | 3.4 Hz | 2.6 Hz |
| **16** | 3.4 Hz | 2.9 Hz |
| **17** | 3.2 Hz | 3.5 Hz |
| **18** | 3.2 Hz | 3.9 Hz |
| **19** | 1.8 Hz | 2.2 Hz |
| **20** | 1.4 Hz | 6.6 Hz |
| **21** | 1.9 Hz | 1.1 Hz |
| **22** | 3.4 Hz | 6.1 Hz |

The table shows the numeric values comparing the dominant LFP frequency and the frequency-tuning value for each neuron, represented visually in Figure 6A. The figure and table display units with increased firing rate at a specific frequency (n=22/27). The units with frequency-specific suppression in firing rates (n=5/27) are not shown.
