# Supplementary figures and images for: Human neuronal firing varies with the frequency of local field potential oscillations
Source: PLoS Biol. 2026 Jun 23;24(6):e3003818. doi: 10.1371/journal.pbio.3003818 (PMC13289887; doi:10.1371/journal.pbio.3003818)

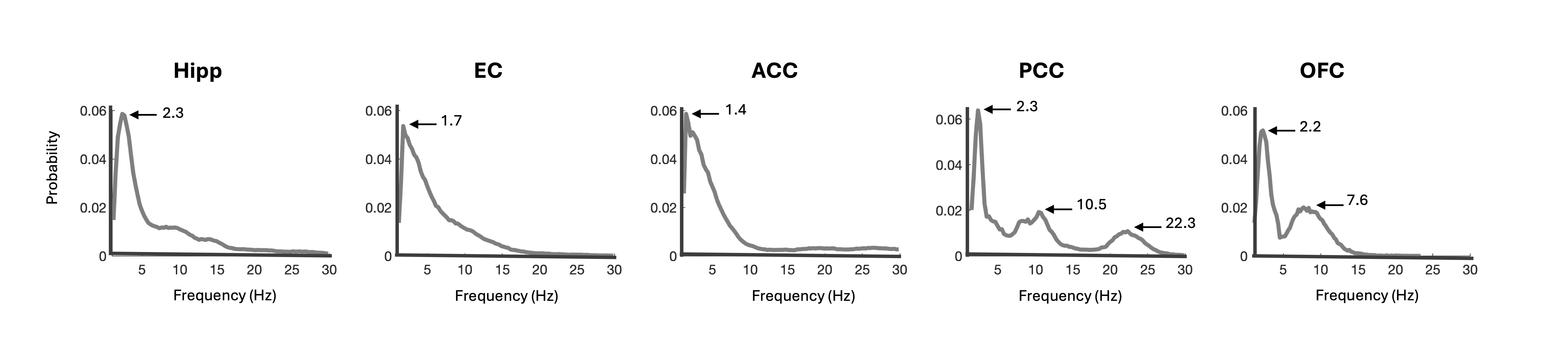

Supplement: S1 Fig — All regions exhibit a dominant peak in the low-theta range (<4 Hz). Additionally, PCC and OFC signals display secondary peaks. Hipp peak at ~2.3 Hz; 73% of the frequency content below 30 Hz falls under 10 Hz. EC peak at ~1.7 Hz; 80% of frequencies <30 Hz are < 10 Hz. ACC peak at ~1.4 Hz; 75% of frequencies <30 Hz are < 10 Hz. PCC peaks at ~2.3 Hz, 10.5 and ~9 Hz; 60% of frequencies <30 Hz are < 10 Hz. OFC peaks at ~2.2 Hz and ~7.6 Hz; 84% of frequencies <30 Hz are < 10 Hz. (TIFF) [file pbio.3003818.s004.tiff]

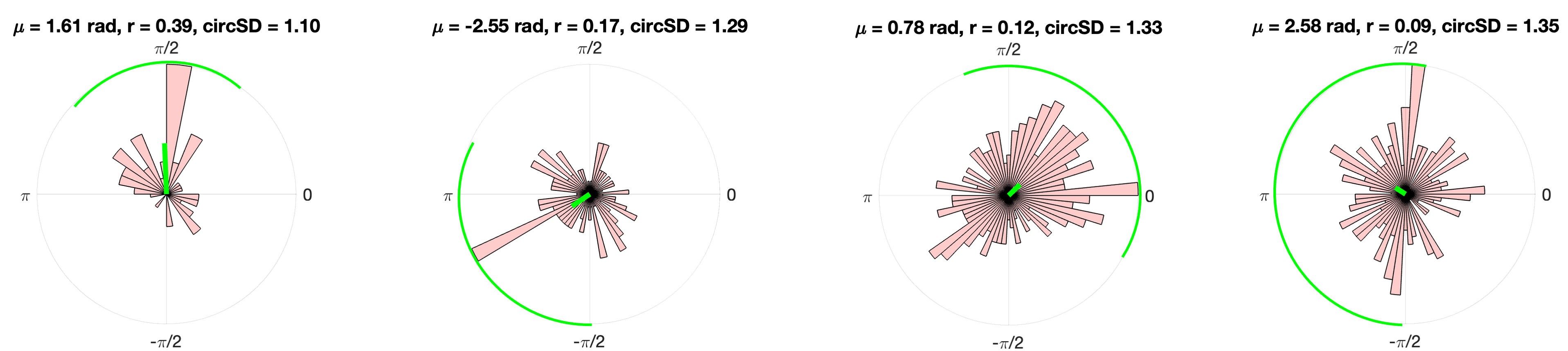

Supplement: S2 Fig — Polar histograms illustrate spike phase distributions for representative neurons, arranged from left to right by decreasing mean resultant vector length (r), corresponding to progressively broader phase dispersion. The histograms show the distribution of spikes across the LFP oscillatory cycle. The green line indicates the mean resultant vector, and the green arc represents the circular spread of phases contributing to significant phase locking (Rayleigh test). These examples showcase how larger r values correspond to narrower phase tuning, while smaller r values reflect more distributed phase preferences. (TIFF) [file pbio.3003818.s005.tiff]

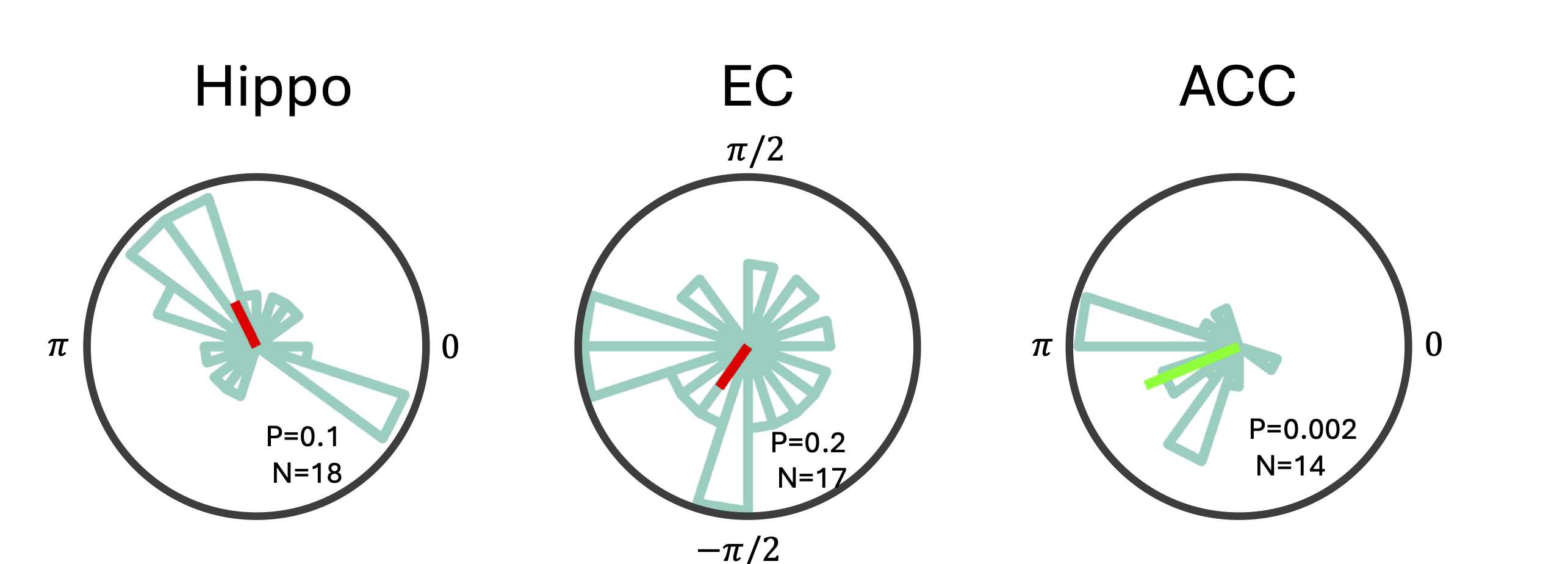

Supplement: S3 Fig — Rayleigh tests revealed no significant phase preference in pooled phase distributions of hippocampal and entorhinal neurons, as indicated by their mean phase vectors (red). In contrast, ACC neurons exhibited a significant preference for the trough of the oscillation, as indicated by the mean phase vector (green). (TIFF) [file pbio.3003818.s006.tiff]

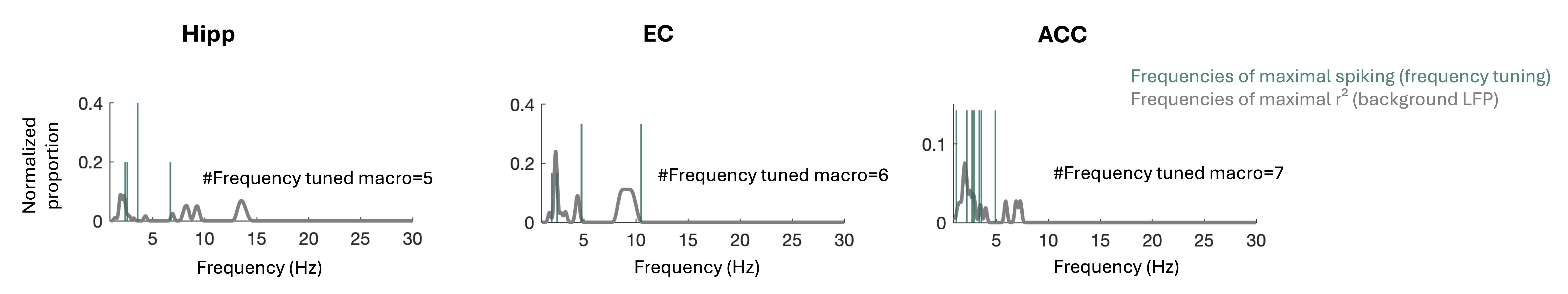

Supplement: S4 Fig — For each region, the pine green bars show the distribution of frequencies at which frequency-tuned neurons exhibited maximal spiking activity. Overlaid in gray is the distribution of frequencies corresponding to the highest r² values across all macroelectrodes in that region, representing the most prevalent background oscillatory frequencies. Across regions, frequency tuning predominantly occurred below 5 Hz and did not consistently align with the dominant peaks of the background LFP distribution. (TIFF) [file pbio.3003818.s007.tiff]

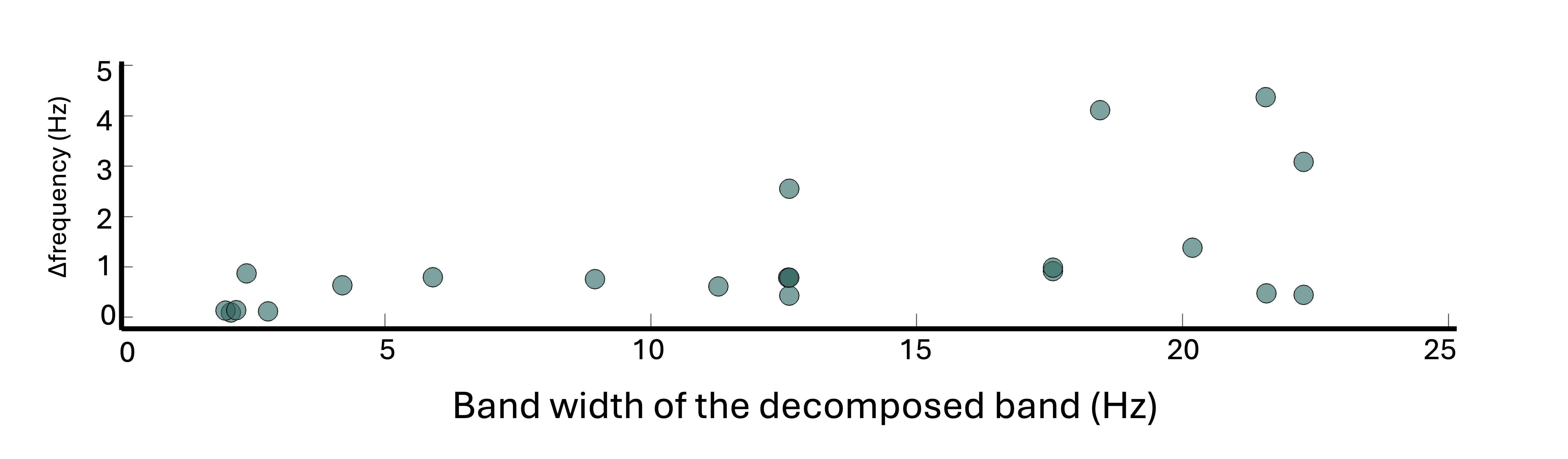

Supplement: S5 Fig — The x-axis shows the bandwidth of each ORCA-decomposed band (upper frequency − lower frequency), while the y-axis plots Δfrequency: the absolute difference between the frequency at which significant tuning is observed and the frequency that maximizes the model fit (peak r²). The narrow spread of Δfrequency values across a range of bandwidths indicates fine resolution in the frequency tuning process. (TIFF) [file pbio.3003818.s008.tiff]

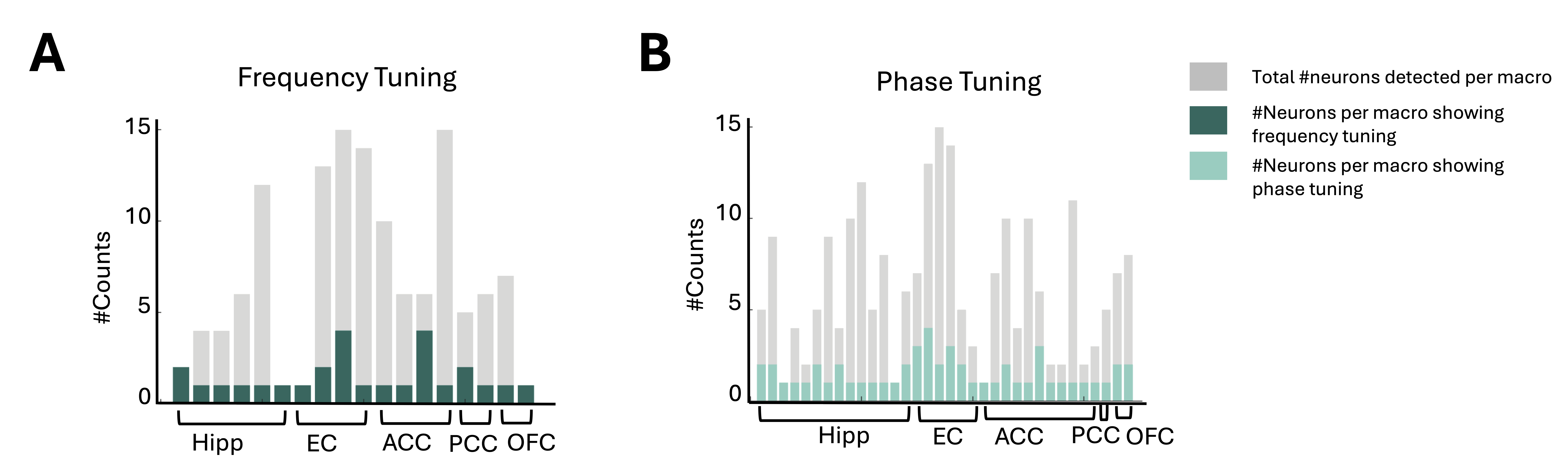

Supplement: S6 Fig — A. Proportion of single-neurons exhibiting frequency tuning, calculated relative to the total number of isolated single-neurons from microwires stemming from the tip of the same macroelectrode. Data are shown across regions for 18 macroelectrodes from which at least one corresponding single neuron exhibited frequency tuning. B. Proportion of single neurons exhibiting phase tuning, relative to the total number of isolated single neurons from microwires stemming from the tip of the same macroelectrode. Data are shown across regions for 34 macroelectrodes from which at least one corresponding single neuron exhibited phase tuning. (TIFF) [file pbio.3003818.s009.tiff]

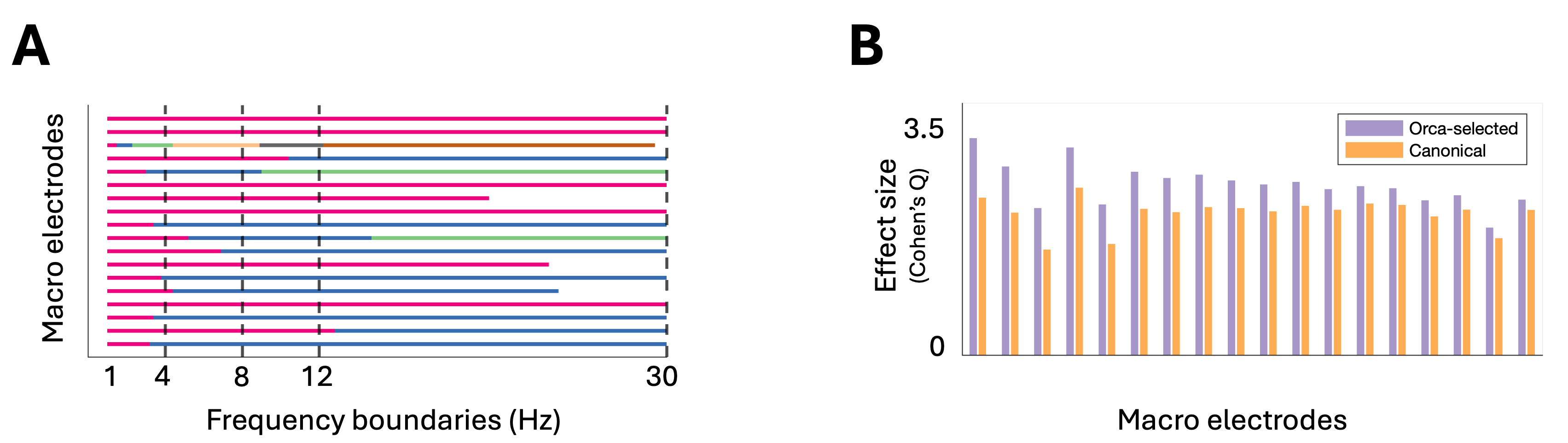

Supplement: S7 Fig — Channels are ordered by descending Cohen’s Q difference, based on ORCA-derived decompositions with a variable number of bands. Colors denote individual bands: pink (first), blue (second), green (third), orange (fourth), gray (fifth), and brown (sixth). Most electrodes (83%) were decomposed into one or two bands. Dashed lines indicate the boundaries defined by canonical frequency bands. Notably, these boundaries do not align with the ORCA-derived bands, highlighting differences between data-driven and canonical approaches. B. Comparison of reconstruction quality between ORCA and classical methods across channels, illustrating the improvement in fit achieved by ORCA, quantified by Cohen’s Q difference. Across all channels, Cohen’s Q for the ORCA-selected method consistently exceeded that of the canonical approach, with differences ranging from 0.16 to 0.94. The color scheme matches Fig 2, with purple indicating the ORCA-selected method and orange denoting the classical method. (TIFF) [file pbio.3003818.s010.tiff]

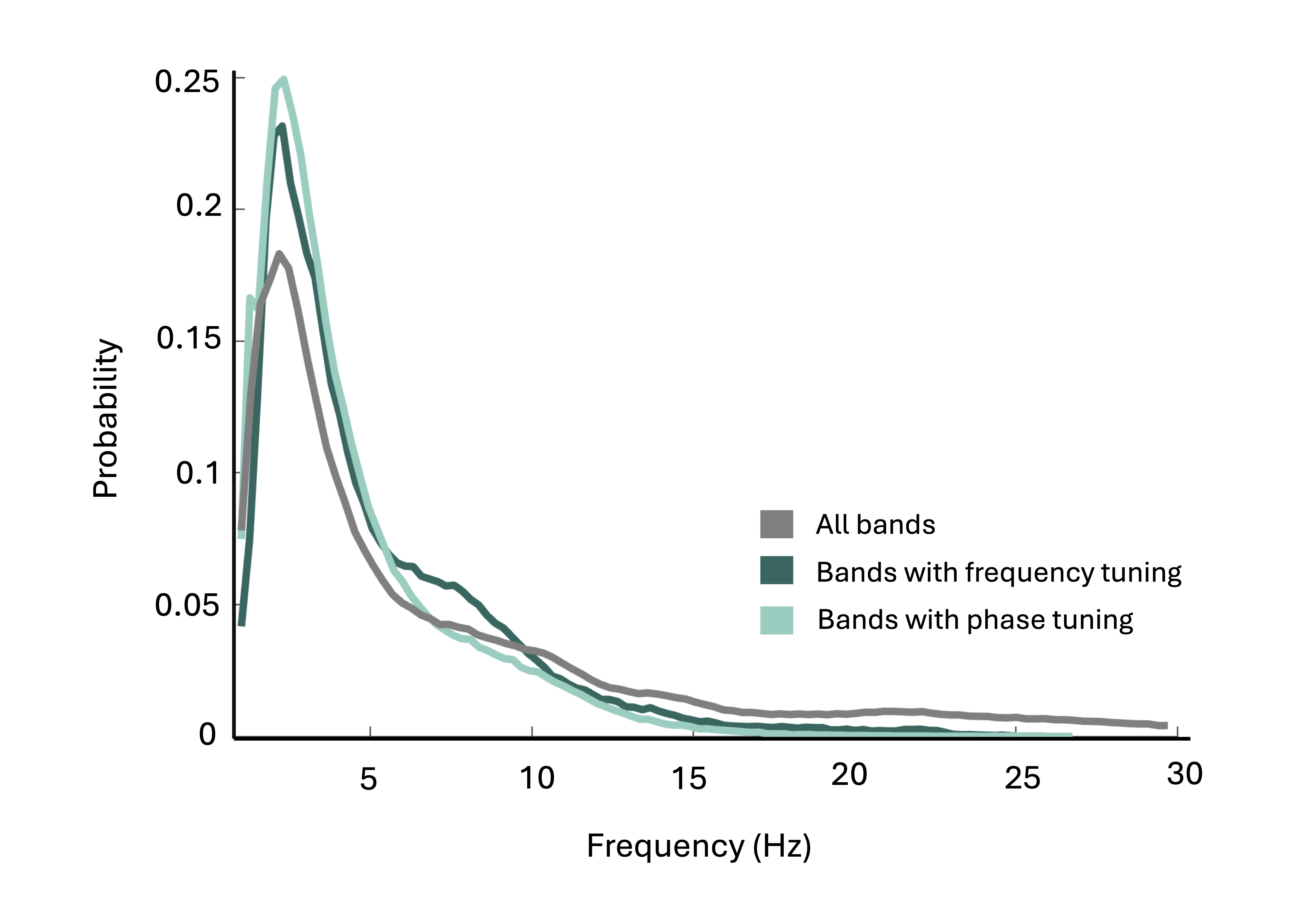

Supplement: S8 Fig — Both phase and frequency tuning preferentially occur at low theta frequencies, resulting in tuning-specific distributions that differ markedly from the overall frequency distribution. (TIFF) [file pbio.3003818.s011.tiff]
