# Supplementary material for: Human neuronal firing varies with the frequency of local field potential oscillations
Source: PLoS Biol. 2026 Jun 23;24(6):e3003818. doi: 10.1371/journal.pbio.3003818 (PMC13289887; doi:10.1371/journal.pbio.3003818)
Supplement: Attachment — Submitted filename: Frequency_Tuning_ResponseLetter_final.pdf [file pbio.3003818.s012.pdf]

Response to Reviewers

We sincerely thank the reviewers and editor for their enthusiasm and thoughtful evaluation of our manuscript. We have carefully and expansively updated the manuscript to address the essential revisions outlined by the reviewers. We first provide an executive summary of revisions followed by our detailed response to individual reviewer comments.

In the revised version of the manuscript, we include 1 new figure and 2 new tables in the supplemental materials. Supplemental Figure 2 provides examples of narrow and broad phase tuning. New tables provide additional information regarding: 1) the distribution of effects across participants (Supplementary Table 2), and 2) the tuning preference of frequency-tuned neurons along with the predominant LFP frequency detected in the macro recording (Supplementary Table 3). We have also modified two Figures (Figure 1 (we added panel A and modified panel D) and Supplementary Figure 6) for enhanced visual clarity.

Reviewers 1 and 2 raised concerns about the predominance of effects at slow frequencies. Similarly, they asked how the temporal autocorrelation of instantaneous phase and frequency could influence analyses assessing the independence of phase- and frequency-tuning. We performed 2 new suggested control analyses using ISI-shuffling analysis or split-halves. In both analyses, we continue to observe frequency-tuned neurons further reinforcing the robustness of our reported findings. We further tested and excluded the possibility that frequency variability influences our ability to detect phase tuning. These 3 new analyses are now included in the revised manuscript.

Reviewers 2 and 3 requested clarifications on signal referencing and usage of macro-electrode LFP. In brief, our use of the LFP from the adjacent macroelectrode was motivated by our theoretical interest in determining how neuronal firing varies as a function of circuit-level oscillatory activity rather than those extremely local "micro LFP" signals captured via microwires. Consequently, we did not acquire low-pass filtered micro-LFP signals from microwires and so are unable to perform a direct comparison between frequency-tuning to micro vs macro LFPs. We raise this point for future work in the discussion.

Reviewers 2 and 3 reviewers requested further clarification regarding the ORCA method and how it relates to more standard analytical approaches such as the "filter and Hilbert" method. ORCA also uses the "filter and Hilbert" method but does so in frequency bands defined based on a data-driven approach. We now provide further description of how ORCA relates to these approaches and clarify its utility in identifying frequency-tuned neurons in the discussion section.

Below, we provide all reviewer comments (shown in black) along with our response (shown in green) and text that has been added to the manuscript (shown in *blue*).

Reviewer #1: Summary

In their manuscript, "Human neuronal firing is modulated by the frequency of local field potential oscillations", the authors address an important question: how neural oscillations, decomposed into their two fundamental properties (frequency and phase) relate to single-neuron firing across brain regions. To approach this, they leverage a rich multimodal dataset containing both LFP and single-neuron recordings from five regions, offering a valuable opportunity to link region-level and cellular-level dynamics.

The authors report that a subset of neurons (15%) exhibited clear phase tuning, with spiking aligned to specific oscillatory phases. A detailed case example illustrates this phenomenon well, and further analyses confirm that the same neuron shows no preference for instantaneous frequency. Interestingly, ACC neurons demonstrate a higher prevalence of phase tuning. Beyond this, the authors also identify a complementary subset of neurons tuned to instantaneous frequency but not phase, and they classify three distinct types of frequency-related tuning. This dual approach provides a nuanced view of how oscillatory dynamics may guide spiking behavior. However, there're still several points that need to be considered

Major Points for Consideration

1. The manuscript convincingly develops the analysis of frequency tuning, while the treatment of phase effects could be expanded further. Since both phase and frequency are continuous variables, adopting a probability density framework for phase (similar to what was done for frequency) might provide a more balanced and informative comparison. This would be especially valuable because more neurons in the dataset exhibited phase tuning (54/357) compared to frequency tuning (27/357). Exploring phase tuning in greater detail could therefore yield deeper insight and make the study's conclusions even stronger.

Rather than contrasting spiking probability against a uniform phase distribution, the authors could present spiking probability density as a direct function of phase. This would clarify how probability mass is concentrated in preferred ranges and reveal whether spiking is sharply confined to narrow phase windows or more broadly distributed. Such an approach could significantly enrich the story of how phase modulates spiking.

Response: We thank the reviewer for this thoughtful suggestion and agree that a more detailed characterization of phase tuning can further enrich the manuscript. While both phase and instantaneous frequency are continuous variables, they differ in their mathematical structure: phase is a circular variable with a natural uniform null distribution, whereas frequency is a linear, non-periodic variable. For this reason, our primary phase analyses relied on circular statistics designed to test deviations from circular uniformity (Rayleigh test), which is the standard most used null model for evaluating phase locking in the prior studies. To address the question raised by the reviewer, we expanded our analysis: beyond testing for non-uniformity using Rayleigh's test; we characterized phase tuning strength using the mean resultant length (r), which measures the concentration of probability mass around a preferred phase. This provides a direct analogue to the frequency-domain distributional analysis, capturing whether spiking is confined to narrow phase windows or broadly distributed across phase. We quantified the concentration of phase probability mass around a preferred phase (*circ_r* function, Matlab Circular Statistics Toolbox), providing a continuous measure of phase-locking strength with larger r values indicating that spiking probability density is narrowly concentrated within a restricted phase range, whereas smaller values reflect broader phase distributions.

Based on r values, phase-tuned responses were categorized into two groups: relatively broad phase distributions ($r < 0.1$), relatively narrow phase tuning ($r \geq 0.1$). Among the 54 neurons exhibiting significant phase modulation, 24 fell into the relatively broad distribution group, 30 showed relatively narrow phase tuning.

To provide an interpretable measure of phase dispersion, we additionally computed the circular standard deviation of spike-associated phases (*circ_std* function). Across phase-tuned neurons, circular standard deviation ranged from 1.10 to 1.39 radians (63.2°–79.6°), indicating that spiking probability was generally distributed over approximately 17–22% of the oscillatory cycle. Together, these results suggest that in neurons showing statistically significant phase modulation, spiking was typically biased toward a relatively narrow phase range.

We added this paragraph to the manuscript to the **Result section** under **A Subset of Human Single Neurons Displays Phase Tuning**:
Inspection of the mean resultant vector (r) and of the circular standard deviation measurements further indicated that spike probability was typically (55%) confined to relatively narrow phase tuning ($r \geq 0.1$) and the dispersion of the spikes was distributed over approximately 17–22% of the oscillatory cycle (see ***Supplementary Figure 2****).*

We have further included this in the **Methods section** under **Statistical analyses**:
Beyond testing for non-uniformity using Rayleigh's test, we quantified the strength of phase tuning using the mean resultant vector length (r), which measures the concentration of phase-associated spiking probability around a preferred phase. Larger r values indicate stronger, more narrowly concentrated phase locking, whereas smaller values reflect relatively broader phase modulation. Based on r, phase-tuned responses were categorized into two groups: relatively broad phase distributions ($r < 0.1$), and relatively narrow phase tuning ($r \geq 0.1$). We also computed the circular standard deviation to further evaluate the dispersion of the spikes across the oscillatory cycle. Circular statistical analyses were performed using the CircStat2012a MATLAB toolbox (circ_r and circ_std respectively).

We have further included this in the **Supplementary materials**:
Supplementary Figure 2. *The figure shows examples of phase tuning with varying dispersion.*
Polar histograms illustrate spike phase distributions for representative neurons, arranged from left to right by decreasing mean resultant vector length (r), corresponding to progressively broader phase dispersion. The histograms show the distribution of spikes across the LFP oscillatory cycle. The green line indicates the mean resultant vector, and the green arc represents the circular spread of phases contributing to significant phase locking (Rayleigh test). These examples demonstrate that larger r values correspond to narrower phase tuning, while smaller r values reflect more distributed phase preferences.

2. Clarifying independence of phase and frequency tuning
The manuscript makes an important contribution by showing that phase tuning and frequency tuning are independent phenomena, likely reflecting distinct mechanisms. One challenge, however, is that instantaneous frequency and phase are not strongly correlated by definition: phase evolves continuously, while instantaneous frequency does not. This inherent difference may reduce the chance of detecting neurons tuned to both properties under the current framework.

It would strengthen the work to clarify how this methodological issue is accounted for, or to briefly discuss alternative approaches that might better capture potential overlap between frequency and phase tuning. Even acknowledging this limitation would help readers interpret the results more accurately.

Response: We thank the reviewer for this thoughtful and constructive comment. We agree that clarifying the relationship between instantaneous phase and instantaneous frequency is important for interpreting the dissociation between phase tuning and frequency tuning.

The method used in our work (ORCA) relies on its primary contribution that is its *data-driven identification and validation of frequency bands* through signal reconstruction. Once these band boundaries are established, instantaneous phase and frequency are estimated using standard analytic-signal methods (i.e., band-limited filtering followed by the Hilbert transform). Instantaneous frequency is computed as the temporal derivative of the unwrapped Hilbert phase, consistent with analytic-signal theory.

As pointed out by the reviewer, although phase and instantaneous frequency are mathematically related, they exhibit fundamentally different statistical structures and temporal dynamics in practice. Phase is a bounded, circular variable that evolves continuously on a cycle-by-cycle timescale, whereas instantaneous frequency is a linear quantity that typically varies more slowly and shows stronger temporal autocorrelation. This distinction is evident in our data (Fig. 1D), where frequency fluctuations occur over longer timescales than phase fluctuations. As a result, phase tuning is preferentially sensitive to precise spike timing within individual oscillatory cycles, whereas frequency tuning likely reflects slower, more sustained network states.

Importantly, this temporal asymmetry implies that the low co-occurrence of phase- and frequency-tuned neurons is unlikely to arise from a methodological artifact inherent to their computation. Rather, it reflects the fact that phase and frequency index different aspects of oscillatory dynamics operating on distinct timescales. While instantaneous frequency is derived from phase, the two quantities are not required to be strongly correlated in empirical data, particularly in nonstationary neural signals. Thus, the observed dissociation between phase and frequency tuning supports the interpretation that these measures capture separable coding mechanisms, rather than reflecting a computational bias.

Finally, we acknowledge that alternative analytical frameworks—such as cycle-resolved waveform analyses that explicitly characterize oscillations based on their shape and asymmetry (e.g., the ByCycle approach; Bradley Voytek and Scott Cole, 2017)—may capture oscillatory structure differently. Such approaches could, in principle, be better suited to detecting neurons that jointly encode phase- and frequency-related features. These methods address complementary questions and represent an important direction for future work.

In order to raise this important point, we added this paragraph to the **Future Directions** section of the **Discussion** in our manuscript:
Fourth, a limitation of the present study is that instantaneous phase and instantaneous

frequency, while mathematically related, reflect different statistical properties and temporal scales of neural signals. Phase captures rapid cycle-level dynamics, whereas instantaneous frequency varies more slowly and reflects changes in local oscillatory timescale or network state. This asymmetry may reduce sensitivity to neurons that jointly encode phase and frequency within short temporal windows. In addition, although ORCA provides a data-driven and reconstruction-validated framework for estimating instantaneous frequency, it is not designed to explicitly detect or label sustained oscillatory bouts. Consequently, transient or nonsinusoidal signal features may produce local instantaneous frequency estimates that reflect momentary waveform structure rather than persistent oscillations. Instantaneous frequency should therefore be interpreted primarily as a descriptor of local oscillatory timescale rather than direct evidence of sustained sinusoidal rhythms. Future work integrating data-driven band identification with oscillation-detection or cycle-by-cycle waveform-based approaches (e.g., bout-based or morphology-based analyses such as the ByCycle framework (Scott Cole and Bradley Voytek, 2017)) may help clarify how neurons jointly encode phase, frequency, and waveform features across timescales.

3. Distribution of tuning across frequency ranges

The authors do an excellent job showing that frequency tuning is not simply a byproduct of spectral density. A similar level of care could be applied to one of their central claims: that 90% of phase- or frequency-tuned neurons occur at <10 Hz. This is an intriguing result, but it may be partly driven by bias if the LFP spends disproportionately more time in lower frequency ranges.

To strengthen this claim, the authors could quantify the proportion of time the LFP spends in each frequency band. Additionally, computing spike probability relative to the baseline prevalence of each frequency would test whether neurons fire disproportionately at certain frequencies, independent of how much time the signal spends there. This would help confirm whether the bias toward <10 Hz reflects genuine tuning or simply frequency distribution properties.

Response: We appreciate the reviewer's point regarding potential occupancy bias. Because ORCA returns instantaneous frequency at each timepoint, the normalized instantaneous-frequency histograms we reported already quantify frequency occupancy (i.e., the proportion of time the LFP spends at each frequency; also Figures 1 and 3-5). Importantly, our frequency-tuning statistic is explicitly referenced to this baseline occupancy: tuning is detected by comparing the LFP frequency distribution at spike times to the baseline LFP instantaneous-frequency distribution within the same ORCA-defined band. Thus, a neuron can only be classified as frequency-tuned if spikes occur disproportionately at a given LFP frequency relatively to the baseline prevalence of that LFP frequency. Consistent with this, we find that preferred (spike-associated) frequencies do not systematically coincide with the modal/most prevalent LFP frequency, indicating that tuning is not a passive consequence of dwell time in dominant frequencies. Below we show the numeric values of Figure 6A in a table format (Figure 6A x and y axis names in italics for reference). This shows the frequency-tuned units displaying an increased firing rate at a given frequency (n=22/27). The frequency-tuned units displaying a selective suppression of firing within a frequency range

(n=5/27) are not shown. We now have included this table in supplementary materials (new Table S3).

**Neuron**	**Dominant LFP frequency (Hz)**<br>***y axis: Frequency at max R^2***	**Preferred tuning frequency (Hz)**<br>***x axis: Frequency at increased spiking***
**1**	7.8 Hz	9.2 Hz
**2**	7.8 Hz	8.8 Hz
**3**	1.7 Hz	2.1 Hz
**4**	2.4 Hz	3.6 Hz
**5**	2.8 Hz	3.4 Hz
**6**	4.2 Hz	2.4 Hz
**7**	4.2 Hz	4.6 Hz
**8**	4.2 Hz	4.9 Hz
**9**	2.9 Hz	2.5 Hz
**10**	8.4 Hz	10.4 Hz
**11**	3.7 Hz	1.8 Hz
**12**	1.7 Hz	10.1 Hz
**13**	3.4 Hz	4.9 Hz
**14**	3.4 Hz	3.6 Hz
**15**	3.4 Hz	2.6 Hz
**16**	3.4 Hz	2.9 Hz
**17**	3.2 Hz	3.5 Hz
**18**	3.2 Hz	3.9 Hz
**19**	1.8 Hz	2.2 Hz
**20**	1.4 Hz	6.6 Hz
**21**	1.9 Hz	1.1 Hz
**22**	3.4 Hz	6.1 Hz

We also added a paragraph to the **Discussion section** of the manuscript under **A subset of human single neurons displays frequency-tuning to slow oscillations independent of the phase of oscillation**:
Neurons were classified as frequency-tuned only when spikes occurred disproportionately relative to the baseline prevalence of LFP oscillatory frequencies, ensuring that the predominance of tuning below 10 Hz cannot be explained by the LFP spending more time at lower frequencies. Notably, preferred spike-associated frequencies did not systematically coincide with the modal LFP frequencies, further indicating that the predominance of tuning below 10 Hz cannot be explained by the greater prevalence of low-frequency activity (Supplementary Table 3).

Minor Points
1. Including multiple axis views of the transparent brain in Figure 1A would make anatomical localization of recording sites clearer.

Response: We thank the reviewer for the comment we included all 3 views of the brain to the Figure 1C. Images are copied below for convenience.

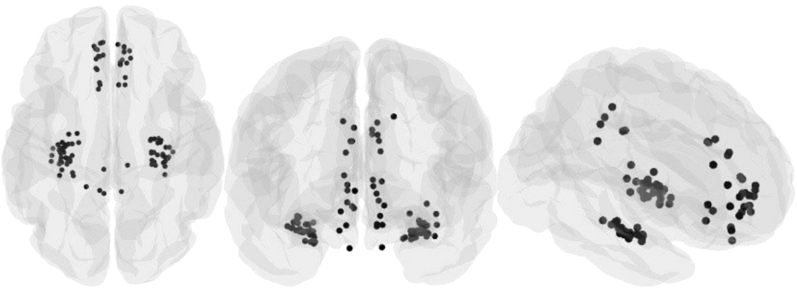

2. In Figure 1B, using a more intuitive color scheme (e.g., green for preferred phase ranges, grey otherwise) would highlight phase tuning more clearly. A similar approach could be applied to distinguish frequency-tuned from non-tuned neurons.

Response: We thank the reviewer for this comment. To improve the readability of the panel (now Figure 1D), we modified the phase-tuned panel (left): the spike times are color-coded according to the LFP phase at which they occurred, similar to the frequency-tuned panel (right). We also revised the caption to further clarify the figure.

3. The "two-way KS test" would be better described as a "two-sample KS test."

Response: Thank you, we have now changed this in the revised version.

Reviewer #2: The authors investigate a fairly novel question using a very rare dataset: does the timing of human neurons align with the instantaneous frequency of the local field potential (LFP)? Given the relatively high variance in frequency that human LFPs exhibit,

particularly in theta oscillations in the medial temporal lobe, this study is well posed to provide valuable information to researchers interested in temporally-mediated information encoding in the human brain. There are several issues that the authors should address, though, before the manuscript is ready for publication:

1. I am concerned be the overall predominance of very slow frequencies (1-2.5 Hz; Supplementary Figure 1), and implications this may have, since a large proportion of the small # of neurons showing any significant effect are within the 1-5 Hz range. I wonder if oscillations in this range are more likely to yield significant phase- and frequency- locking estimates due to the high autocorrelation in such slow signals. I don't think the circular shuffle fully addresses this possibility. Let us imagine a neuron whose firing rate ramps up slowly over the 5-minutes of recording time. At the same time, imagine that the LFP instantaneous frequency gradually decreases from 3 Hz to 2 Hz. Now, spikes will sample the low-frequency epochs more heavily, so the empirical spike-frequency histogram skews low. If you circularly shift the frequency trace, the ramp-up in firing will now align with higher or lower parts of the frequency trace arbitrarily, which will intrinsically result in lower spike-frequency coupling in the shuffled distribution than the empirical data. But that would not necessarily support the idea that spikes are "tuned" to low-frequencies, but just that the two variables systematically drifted in opposite directions slowly over time. I would bet this pattern is more likely in data that is predominantly very slow, as is the case in this dataset. The authors could attempt to shuffle the spike times themselves, using an ISI-based shuffling approach, or even show that the effects are correlated across time using a split-half analysis.

Response: We thank the reviewer for this important comment. We deliberately avoided random shuffling of frequency samples because it destroys the slow temporal structure intrinsic to low-frequency oscillations, yielding an unrealistic null hypothesis. Circular shifting preserves this slow structure while disrupting spike–frequency alignment, making it a more appropriate primary control for slow oscillatory signals. However, we agree that circular shifting alone may not fully address a scenario in which firing rate and instantaneous frequency co-drift slowly over time, which motivated our additional analysis as a complementary and more conservative control. To directly test this possibility, we implemented the suggested analysis using ISI-preserving spike-time shuffle as a more conservative control. This procedure maintains the spike-train's firing-rate profile and inter-spike interval statistics while destroying its temporal alignment to the instantaneous frequency trace, thereby explicitly breaking slow co-drift between spikes and frequency. Under this control, the majority of frequency-tuned neurons (19/27) remained significant, indicating that the predominant tuning effects cannot be explained by slow temporal co-variation alone. Importantly, ISI-shuffling reduced sensitivity for a subset of neurons (8/27). However, these neurons continued to exhibit stable tuning structure over time: preferred frequencies were highly consistent across recording halves (median Δpref = 0.20 Hz; 25th–75th percentile: 0.09–0.50 Hz), and spike-sampled frequency distributions were strongly correlated across halves (median Spearman rho = 0.8). Together, these analyses demonstrate that while ISI-shuffling provides a stringent robustness check against slow co-drift, the

observed frequency-tuning effects are stable in structure and cannot be solely attributed to gradual temporal drift.

In order to improve and strengthen the quality of the work we have added this control analysis in the **Results section** under **A Different Subset of Human Single Neurons Displays Frequency Tuning**:
We performed several control analyses to determine whether frequency tuning was a trivial byproduct of slow co-variations between firing rate and instantaneous frequency. First, we implemented an ISI-preserving spike-time shuffle, which maintains the spike train's firing-rate profile and inter-spike interval statistics while disrupting its temporal alignment to instantaneous frequency. These analyses were used as robustness checks. Using this procedure, we found that the majority of neurons (19/27) remained significant. Second, using a split halves analysis, we observed that frequency preference was temporally stable: preferred frequencies differed minimally across recording halves (median Δpref = 0.20 Hz; 25th–75th percentile: 0.09–0.50 Hz), and spike-sampled frequency distributions were highly correlated across halves (median Spearman rho = 0.8). Thus, frequency tuning cannot be attributed to gradual temporal drift and was reliably detected across recording segments. This temporal stability argues against a monotonic drift explanation for the observed tuning effects.

2. It is possible that I am misunderstanding how instantaneous frequency is extracted by ORCA, but it appears that very little information is provided about the amplitude of the signal at frequencies that are supposedly tuning spiking. The relationship of frequency tuning to the power spectrum should be explored (similar to Figure 6). Is there a relationship (or bias) between frequency estimates and power? I think a more convincing analysis would stratify frequency-tuning by instantaneous amplitude/power bins (e.g., low/medium/high) and test frequency tuning within each bin.

Response: The reviewer raises an interesting point about the influence of amplitude, not explored in our original submission. To evaluate whether frequency tuning could reflect a bias toward high or low amplitude epochs, we stratified instantaneous frequency estimates according to instantaneous amplitude and repeated the frequency-tuning analysis within each amplitude regime (similar to Jacobs 2007). Because analyses using three amplitude bins would likely have been underpowered given the spike counts of some neurons, we adopted a median split, yielding low- and high-amplitude epochs with sufficient spikes in each condition.

Of the 27 neurons previously identified as frequency tuned, 16 remained significant in both amplitude regimes, indicating that frequency tuning is expressed independently of instantaneous amplitude. Six neurons exhibited tuning in only one amplitude regime, and five neurons were not significant after stratification (likely because spike counts were not sufficient once separated in the two groups). Notably, tuning was not restricted to high-amplitude epochs, arguing against the possibility that frequency tuning arises from a trivial bias toward high-power periods.

This variability likely reflects differences in the underlying baseline frequency distributions within each amplitude regime rather than a systematic power-driven effect. The distribution of instantaneous frequencies during spiking among the two regimes were not statistically different for all the 27 neurons. Together, these results indicate that neurons can show tuning to a specific LFP frequency regardless of its amplitude variations.

We added this to the **Results section** under **A Different Subset of Human Single Neurons Displays Frequency Tuning**:
Prior work suggests that oscillatory phase and amplitude play complementary roles in organizing neuronal activity. Importantly, Jacobs et al. demonstrated that the strength of phase-dependent firing varies with oscillatory power, such that neurons exhibit stronger phase modulation during periods of higher oscillatory amplitude (Jacobs et al., 2007). More recent intracranial human recordings have similarly shown that spike–phase locking becomes more precise during high-power oscillatory epochs, indicating that oscillatory amplitude can enhance the strength of phase tuning without necessarily altering the preferred phase itself (Guth et al., 2025).
We therefore examined whether frequency tuning was related to oscillatory amplitude, by repeating the frequency tuning analysis after stratifying instantaneous frequency estimates by instantaneous amplitude. We used a median split to define low- and high-amplitude "regimes" and evaluated frequency tuning independently within each regime. Of the 27 neurons previously identified as frequency tuned, 16 remained significant in both amplitude regimes, six showed significant tuning in only one regime, and five were not significant after stratification, perhaps due to reduced spike counts within the amplitude subsets. Importantly, tuning was not restricted to high-amplitude regimes. Together, these results indicate that frequency tuning can occur even during low-amplitude regimes and thus does not critically depend on amplitude fluctuations.

3. The authors show that phase-locked cells do not overlap with frequency-locked cells. But do phase-distributions systematically differ in recordings with high instantaneous frequency variance, in a way that might systematically prevent the identification of simultaneous phase- and frequency- locking?

Response: We thank the reviewer for this important methodological point; instantaneous phase and instantaneous frequency are characterized by distinct statistical properties and temporal scales. Phase captures rapid, cycle-level dynamics, whereas instantaneous frequency varies more slowly and reflects longer-lasting network states. This asymmetry may reduce sensitivity to neurons that jointly encode phase and frequency within short time windows (please also see our response to Reviewer 1.2 as it relates to the overlap of phase- and frequency-tuned neurons).
To test whether elevated instantaneous-frequency variability could impair phase estimation and thereby reduce our ability to detect phase locking, we performed a time-resolved control analysis within frequency-tuned neurons. Instantaneous-frequency variability instability was quantified using a robust measure of short-timescale frequency jitter. To quantify

instantaneous-frequency variability, we derived a time-resolved frequency jitter metric from the instantaneous frequency trace. Jitter was defined as the absolute sample-to-sample change in instantaneous frequency (i.e., |ΔIF|), which captures rapid fluctuations in frequency rather than slow drifts. This signal was smoothed over a short temporal window to reduce noise and yield a local estimate of frequency variability instability. Time points were then divided into low- and high-variability epochs based on the median of this distribution. Phase-locking strength (mean resultant vector length) was then computed (*circ_r* function) separately for spikes occurring in each epoch and compared within neurons. Phase-locking strength did not differ systematically between low- and high-variability epochs (signrank test, $p = 0.44$), indicating that elevated instantaneous-frequency variance does not impair detection of phase locking in our data. These results argue against the dissociation between phase- and frequency-locked neurons arising from a methodological bias related to frequency variability.

We add this paragraph as the last paragraph in **Results** under **Phase and Frequency Tuning are Independent Phenomena** section:
Only 1% of neurons (3/357) were significantly modulated by both the instantaneous frequency and phase of the local LFP, indicating minimal overlap between these two forms of tuning. This low co-occurrence suggests that frequency and phase tuning largely reflect distinct neuronal response properties rather than general characteristics of the local field signal at individual recording sites. Because instantaneous phase and instantaneous frequency are mathematically related, we tested whether variability in instantaneous frequency could bias phase estimation and thereby reduce the ability to detect phase tuning. Within frequency-tuned neurons, we quantified short-timescale frequency instability using a time-resolved frequency jitter metric derived from the instantaneous-frequency trace (defined as the absolute sample-to-sample change in instantaneous frequency, |ΔIF|). Time points were divided into low- and high-variability epochs based on the median jitter value, and phase-locking strength (mean resultant vector length) was computed separately for spikes occurring within each epoch. Phase-locking strength did not differ between low- and high-variability epochs (signrank test, $p = 0.44$), indicating that elevated instantaneous-frequency variability did not impair detection of phase tuning. These results argue against the dissociation between phase- and frequency-tuned neurons arising from a methodological bias related to frequency variability.

4. I think it's interesting but somewhat hard to interpret that the authors only utilize the closest macro-electrode contact for their analyses. Most prior work uses the LFP recorded from the microwire (making it more truly "local"). How does this choice affect the authors results? How does the use of a common-average reference affect the results (compared to an arguably more local bipolar reference)?

Response: We thank the reviewer for this comment and for allowing us to expand on this important choice. Our use of the LFP from the adjacent macrocontact was motivated by our theoretical interest in determining how neuronal firing varies as a function of circuit-level oscillatory activity rather than those extremely local “micro LFP” signals captured via microwires. Consequently, we did not acquire low-pass filtered micro-LFP signals from

microwires and we would be unable to perform a comparison between frequency-tuning to micro vs macro LFPs.

Regarding referencing, we employed common-average referencing (CAR) to enhance oscillatory signals that are shared across contacts and to suppress channel-specific noise. Because our analyses focus on frequency tuning and phase preference relative to dominant rhythms, preserving coherent oscillatory structure is critical. Bipolar referencing, while more spatially local, can attenuate globally coherent oscillations, distort phase relationships, and introduce reference-dependent frequency artifacts—effects that could reduce sensitivity to the population-level oscillatory features central to our study.

We raise this point for future work in the **Discussion**, under **Future Directions**:
Fifth, an important consideration is the spatial scale of the field potential used in the present analyses. The LFP recorded from the nearest macro-electrode contact should be interpreted as a proxy for local network activity rather than a strictly local field potential at the microwire tip, as macro contacts integrate neural signals over a spatial scale of several millimeters. Accordingly, the frequency tuning described here reflects relationships between single-neuron spiking and population-level oscillatory dynamics rather than much more local fields. Future work comparing microwire-local LFP signals on matched datasets will help clarify how frequency tuning manifests across spatial scales.

5. Could "changes in instantaneous frequency" be explained by non-sinusoidal changes in waveform shape?

Response: We thank the reviewer for this question. We agree that nonsinusoidal waveform features and transient events can, in principle, influence instantaneous frequency (IF) estimates when IF is defined as the temporal derivative of Hilbert phase. This limitation applies to all phase-based IF estimators and is well documented in the literature. Importantly, ORCA does not claim to detect only “true,” sustained sinusoidal oscillations, nor does it assume that oscillatory structure is continuous or stationary. Instead, ORCA provides a data-driven framework for extracting and validating spectral representations based on their ability to explain the observed signal.
Cycle-by-cycle and bout-based methods such as bycycle address a complementary question. These approaches explicitly detect oscillatory bouts and quantify waveform morphology (e.g., rise–decay or peak–trough asymmetry) on a per-cycle basis, making them well suited for studying nonsinusoidal waveform shape when that is the primary scientific focus. Future work could integrate ORCA-derived timescale estimates with explicit cycle-based waveform metrics to further dissociate timescale modulation from waveform morphology.

We included a paragraph expanding on this important point in **Discussion**, **Future Directions**. The text is reported above in response to Reviewer 1, comment 2.

6. I am having a hard time parsing Supplementary Figure 6.

Response: We acknowledge that the original version of Supplementary Figure 6 was difficult to interpret. We revised the figure (now Supplementary Figure 7) and improved the caption to explicitly define what each panel represents.

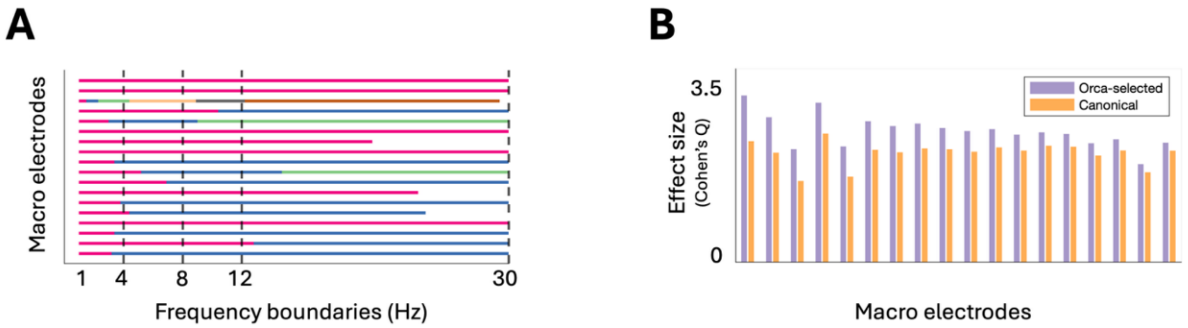

Supplementary Figure 7. A. *Frequency boundaries for 18 macro electrodes in which single neurons exhibited significant tuning to the local field potential (LFP). Channels are ordered by descending Cohen's Q difference, based on ORCA-derived decompositions with a variable number of bands. Colors denote individual bands: pink (first), blue (second), green (third), orange (fourth), gray (fifth), and brown (sixth). Most electrodes (83%) were decomposed into one or two bands. Dashed lines indicate the boundaries defined by canonical frequency bands. Notably, these boundaries do not align with the ORCA-derived bands, highlighting differences between data-driven and canonical approaches.* ***B.*** *Comparison of reconstruction quality between ORCA and classical methods across channels, illustrating the improvement in fit achieved by ORCA, quantified by Cohen's Q difference. Across all channels, Cohen's Q for the ORCA-selected method consistently exceeded that of the canonical approach, with differences ranging from 0.16 to 0.94. The color scheme matches Figure 2, with purple indicating the ORCA-selected method and orange denoting the classical method.*

7. I recognize the difficulty in accounting for inter-subject differences (e.g. mixed-effects modeling) in a small dataset like this, but it would at least be helpful to know how many significant phase- and frequency- locking neurons are contributed by each individual subject.

Response: We thank the reviewer for this comment, and we agree on the importance of addressing contributions from individual patients with respect to the effects of interest. We generated a novel table (new Table S2) with this information and added it to the **Supplementary materials**.

Table: Distribution of frequency- and phase-tuned neurons across subjects:

Subject	Frequency tuned	Phase tuned	Phase & frequency tuned

	**Macro**	**Unit**	**Macro**	**Unit**	**Macro**	**Unit**
**1**	1	2	2	3	1	1
**2**	2	3	2	3	-	-
**3**	1	1	3	4	-	-
**4**	1	1	1	1	-	-
**5**	-	-	3	5	-	-
**6**	2	3	3	8	-	-
**7**	2	5	3	4	-	-
**8**	1	1	3	7	-	-
**9**	-	-	1	1	-	-
**10**	2	5	-	-	2	2
**11**	1	1	1	1	-	-
**12**	2	2	2	2	-	-
**13**	1	1	-	-		
**14**	-	-	1	1	-	-
**15**	-	-	1	1	-	-
**16**	-	-	1	1	-	-
**17**	1	1	2	2	-	-
**18**	1	1	5	10	-	-

The table summarizes the number of neurons exhibiting significant frequency tuning, phase tuning, or joint phase-and-frequency tuning for each subject. Counts are reported separately for neurons recorded from macroelectrodes and single units. This breakdown illustrates the contribution of individual subjects to the overall pool of tuned neurons reported in the study and demonstrates that the observed effects are not driven by a single subject.

8. The authors refer to frequencies below 10 Hz as "low theta", which is not in concordance with the literature.

Response: We agree and thank the reviewer for pointing out this oversight. We have rephrased this term to “slow oscillations” or “lower frequencies” throughout the manuscript.

9. Given the extremely rare dataset, authors should cite a highly relevant and recent work examining theta-phase locking in human neurons (PMID: 38948829).

Response: We thank the reviewer for suggesting this paper. We find this work highly relevant to our study and in strong conceptual alignment with our findings. Accordingly, we have cited this work in our manuscript both in **Introduction** and **Results** section.

Reviewer #3: The authors ask whether instantaneous frequency of intracranial EEG predicts activity of single units measures with micro-electrodes in human patients undergoing invasive epilepsy monitoring. The authors analyse data from 19 patients at rest, from which they analysed signald from 84 iEEG contacts and 357 units. Their Analyses show that both, frequency tunig and phase tuning of spikes to the intracranial EEG exists. Further, units are either frequency or phase tuned but not both. Both frequency and phase tuning occur in lower frequency bands <10 hz.

I think this paper contributes novel and important findings to the field and helps us to better understand the relationship between slower, local intracranial EEG fluctuations and spiking activity. Especially the finding on frequency but not phase-tuning in a subpopulation of units is novel.

I have no major concern but rather questions for clarifications mainly on methodological aspects. First, The authors use the ORCA as algorithm to extract instantaneous phase and frequency. As this method is central to obtain instantaneous estimates of dominant frequency and phase, I would appreciate a bit more detail on the motivation and benefits of this procedure, especially in light of the current paper. Do the authors think that more traditional (i.e, filter-hilbert in e.g. the theta range) spectral decomposition methods would result in different results and conclusions and why?

Response: We thank the reviewer for this important methodological question and the opportunity to clarify our motivation for using ORCA to estimate instantaneous phase and frequency.

ORCA adaptively segments the broadband signal into frequency bands using multiple data-driven band-identification strategies (including explained variance/CoD, spectral coefficient of variation, 1/f peak-picking, and canonical bands), and selects the band structure that maximizes cross-validated signal reconstruction accuracy (r^2). This approach allows the frequency boundaries used for spectral decomposition to be tailored to the statistical structure of each signal rather than imposed *a priori*.

Once frequency boundaries are identified, spectral features are estimated using the conventional filter-Hilbert analytic-signal frame. For each band, ORCA applies an optimized band-pass filter, computes instantaneous amplitude and phase via the Hilbert transform, and estimates instantaneous frequency using a frequency-sliding approach (Cohen, 2014) with

median filtering. Frequency estimates outside the identified band are masked as NaNs to mitigate the effects of phase slips and transient artifacts, reducing sensitivity to brief phase-derivative excursions commonly produced by sharp or asymmetric transients.

Critically, instantaneous phase and frequency estimates in ORCA are retained only insofar as they contribute to reconstructing the original signal and generalize to held-out data. In contrast, canonical filter–Hilbert approaches return phase and instantaneous frequency estimates regardless of whether they meaningfully reflect underlying signal structure. As a consequence, instantaneous frequency estimates obtained in this manner are strongly constrained by filter design, biased toward the center frequency of the passband, and are more likely to suppress genuine within-band frequency modulation.

To address the reviewer's question of whether our conclusions depend on the choice of decomposition method, we re-ran the full frequency-tuning analysis using a conventional canonical band-pass Hilbert approach. Using Bonferroni correction across two canonical bands (1–4 Hz and 4–8 Hz), 20 neurons met criteria for frequency tuning ($\alpha = 0.025$), with two neurons overlapping with the ORCA-identified frequency-tuned population.

This is expected because the two approaches define fundamentally different baseline frequency distributions and comparison supports. ORCA-derived frequency bands are adaptive and reflect dominant oscillatory components present in the signal, whereas canonical band-pass filtering imposes fixed boundaries that constrain frequency estimates to predefined ranges. Consequently, the spike-associated and baseline frequency distributions being compared differ substantially between methods.

We further illustrate these methodological differences in an updated version of Supplementary Figure 6 (now Supplementary Figure 7). Panel A shows the number of oscillatory components identified by ORCA for the 18 macro contacts that exhibited significant frequency tuning. Within the 1–30 Hz range, 14 of the 18 contacts were decomposed into only one or two oscillatory bands, indicating that ORCA captures a small set of data-driven, local oscillations rather than fragmenting activity into multiple predefined bands. Panel B quantifies how well the decomposed signals preserve the structure of the raw LFP. Using Cohen's Q as a similarity measure between the raw signal and the reconstructed signal, ORCA-derived components consistently showed higher similarity to raw signal than canonical band-pass filtered signals, indicating that ORCA preserves dominant oscillatory dynamics more faithfully while canonical filtering introduces greater distortion by enforcing fixed frequency boundaries.

In this context, ORCA was selected as a principled framework for estimating instantaneous frequency without imposing fixed band boundaries. Our new analyses indicate that our conclusions do not hinge on methodological idiosyncrasies using this approach. The canonical band-pass Hilbert reanalysis demonstrates that spike–frequency relationships can also be detected under conventional frameworks, albeit with distributions reflecting different analytical assumptions. This supports the view that spike–frequency coupling is a genuine phenomenon rather than an artifact of a specific decomposition method.

We also added this in the **Discussion** section for more clarity under **Non-canonical data-driven decomposition of phase and frequency of neural signal** section:
Supplementary Figure 7A illustrates the ORCA-derived band boundaries for the 18 macro contacts that exhibited significant frequency tuning. Within the 1–30 Hz range, 14 of the 18 contacts were decomposed into only one or two dominant oscillatory bands, and the resulting boundaries generally did not overlap with canonical frequency bands. This demonstrates that ORCA captures a small number of local oscillations rather than fragmenting the signal into predefined spectral ranges.

We also added this in the **Methods** section for more clarity under **Spectral properties of neural signals** section:
ORCA is conceptually aligned with traditional filtering approaches, but critically differs in how the frequency bands themselves are defined. As emphasized by de Cheveigné and Nelken (2019), filtering inevitably entails temporal smearing and can introduce distortions or spurious structure if frequency ranges are poorly matched to the underlying signal. ORCA mitigates this risk by optimizing band selection based on data-driven reconstruction accuracy, thereby tailoring the decomposition to the spectral structure present in each recording. This adaptive strategy reduces reliance on arbitrary band definitions and improves the interpretability of phase- and frequency-resolved neural dynamics, while remaining subject to the same fundamental constraints and caveats inherent to any filtering-based analysis.

Another question relates to how local the low frequency oscillations are the authors record from the macro contacts. I think this is important state that the iEEG signal at the closest macro to the micro contacts is merely a proxy for the local field potential for neurons recorded with nearby microwires. One needs to bear in mind that the macro contact is still a few millimeters away from the microcontacts with which the authors record unit activity using BF-electrodes.

Response: We thank the reviewer for this important comment, also raised by Reviewer 2. We included a paragraph on this matter in the **Future directions** of the paper, the added text is reported above in response to Reviewer 2, comment 4.

A related question concerns the choice of reference for the macro-contact - did they authors re-reference the macro contact to second-closest macro on the same BF-electrode to isolate generators near the microwires?

Response: We thank the reviewer for raising this important point which was also raised by Reviewer 2. Regarding referencing, we employed common-average referencing to enhance oscillatory signals that are shared across contacts and to suppress channel-specific noise. Because our analyses focus on frequency tuning and phase preference relative to dominant rhythms, preserving coherent oscillatory structure is critical. Bipolar referencing, while more spatially local, can attenuate globally coherent oscillations, an effect that could reduce

sensitivity to the population-level oscillatory features central to our study. Further details can be found in response to Reviewer 2 comment 4.

Relatedly, the authors could have used lowpass filtered microwire recordings containing the actual local field potential. What was the reasoning for not using the microwire recordings, considering that there are accepted methods for spike removal, especially when estimating lower frequency oscillations in the LFP?

Response: Also for this comment we refer the reviewer to the response to Reviewer 2, comment 4. In brief, our choice of using the macroelectrode for the LFP signals was conceptually motivated, and we did not record the LFP from the microwires (only a high-passed filtered signal later used to extract the spikes). By leveraging the macro-LFP, we aimed at evaluating the effect of "less local field potentials" than those that we would have been able to record from the microwires, offering a proxy of local network activity at a larger spatial scale.

Minor things:
On page 15, the authors state they did not find any bursting neurons. This seems surprising. Do the authors have an explanation for that.

Response: We thank the reviewer for this important question. The absence of bursting neurons in our dataset reflects our methodological controls to ensure that phase- and frequency-tuning results were not confounded by burst-related spike clustering.

Importantly, all analyses were restricted to well-isolated single units using stringent quality criteria, including a clear refractory period (no spikes within 1 ms and <1% of ISIs <3 ms). This reduces the likelihood that multi-unit activity or spike-sorting artifacts contributed to the observed firing patterns.

To quantify burst firing, we analyzed the inter-spike interval (ISI) distribution for each neuron as: short ISIs (<10 ms) and long ISIs (>100 ms). Specifically, we computed burst index (BI), defined as:

$$BI = (N_{short} - N_{long}) / (N_{short} + N_{long})$$

Across the population, neurons exhibited a low prevalence of short ISIs and predominantly negative BI values (median BI = −0.95, range [−1, 0.5]), indicating that spikes rarely occurred in strong bursts. Only a small minority of neurons showed modest elevations in short-interval counts, but none met criteria consistent with strong or sustained burst firing.

These results indicate that the dataset is not dominated by burst activity, and therefore the observed phase- and frequency-tuning effects are not driven by burst-related spike clustering.

To improve the readability of the paper we included this in the **Results** section under **Phase and Frequency Tuning are Independent Phenomena**

Across the population, neurons exhibited a low prevalence of short ISIs and predominantly negative BI values (median BI = −0.95, range [−1, 0.5]), indicating that spikes rarely occurred in burst. Only a small minority of neurons showed modest elevations in short-interval counts, but none met criteria consistent with strong or sustained burst firing. This suggests that the recorded neurons primarily fired in a regular, non-bursting pattern and therefore the observed phase- and frequency-tuning effects are not driven by burst-related spike clustering.

On page 16, bottom Canolty et al. 2012 is cited in text. I guess it should be in numbered format as well.

Response: Thank you for being so attentive! We corrected that.

Line 466: The sentence starting with "Cohen demonstrated lower-frequency…" is odd and might need rephrasing.

Response: We thank the reviewer for this comment and rephrased this paragraph for better readability.

Original: Cohen demonstrated lower-frequency oscillatory environments were shown to reduce spike thresholds, allowing neurons to fire in response to weaker synaptic input (Cohen, 2014). This frequency-dependent modulation of excitability can provide a mechanistic account for our observed frequency tuning to slower oscillatory states in local field potentials.

Updated sentence (**Discussion** section, **From frequency-sliding to frequency-tuning: manifestations of a shared oscillatory mechanism**)

Using biophysically detailed neuron models, Cohen (2014) demonstrated that decreasing the frequency of oscillatory input lowers spike threshold and increases sensitivity to weak synaptic input. This frequency-dependent effect on excitability offers a plausible mechanism for the preferential tuning of spiking activity to slower LFP oscillatory frequencies observed in our data.
