# Supplementary material for: Human neuronal firing varies with the frequency of local field potential oscillations
Source: PLoS Biol. 2026 Jun 23;24(6):e3003818. doi: 10.1371/journal.pbio.3003818 (PMC13289887; doi:10.1371/journal.pbio.3003818)
Supplement: Peer review history

# Editor report: Decision Letter 0

**DOI:** 10.1371/journal.pbio.3003818.r001

**Authors:** Taylor Hart, PhD

11 Sep 2025

Dear Dr Bartoli,

Thank you for submitting your manuscript entitled "Human neuronal firing is modulated by the frequency of local field potential oscillations" for consideration as a Research Article by PLOS Biology.

Your manuscript has now been evaluated by the PLOS Biology editorial staff, as well as by an academic editor with relevant expertise, and I am writing to let you know that we would like to send your submission out for external peer review.

However, before we can send your manuscript to reviewers, we need you to complete your submission by providing the metadata that is required for full assessment. To this end, please login to Editorial Manager where you will find the paper in the 'Submissions Needing Revisions' folder on your homepage. Please click 'Revise Submission' from the Action Links and complete all additional questions in the submission questionnaire.

Once your full submission is complete, your paper will undergo a series of checks in preparation for peer review. After your manuscript has passed the checks it will be sent out for review. To provide the metadata for your submission, please Login to Editorial Manager (https://www.editorialmanager.com/pbiology) within two working days, i.e. by Sep 13 2025 11:59PM.

If your manuscript has been previously peer-reviewed at another journal, PLOS Biology is willing to work with those reviews in order to avoid re-starting the process. Submission of the previous reviews is entirely optional and our ability to use them effectively will depend on the willingness of the previous journal to confirm the content of the reports and share the reviewer identities. Please note that we reserve the right to invite additional reviewers if we consider that additional/independent reviewers are needed, although we aim to avoid this as far as possible. In our experience, working with previous reviews does save time.

If you would like us to consider previous reviewer reports, please edit your cover letter to let us know and include the name of the journal where the work was previously considered and the manuscript ID it was given. In addition, please upload a response to the reviews as a 'Prior Peer Review' file type, which should include the reports in full and a point-by-point reply detailing how you have or plan to address the reviewers' concerns.

During the process of completing your manuscript submission, you will be invited to opt-in to posting your pre-review manuscript as a bioRxiv preprint. Visit http://journals.plos.org/plosbiology/s/preprints for full details. If you consent to posting your current manuscript as a preprint, please upload a single Preprint PDF.

Feel free to email us at plosbiology@plos.org if you have any queries relating to your submission.

Kind regards,

Taylor

Taylor Hart, PhD,

Associate Editor

PLOS Biology

thart@plos.org

# Peer review: Decision Letter 1

**DOI:** 10.1371/journal.pbio.3003818.r002

**Authors:** Taylor Hart, PhD

23 Oct 2025

Dear Dr Bartoli,

Thank you for your patience while your manuscript "Human neuronal firing is modulated by the frequency of local field potential oscillations " was peer-reviewed at PLOS Biology. It has now been evaluated by the PLOS Biology editors, an Academic Editor with relevant expertise, and by several independent reviewers.

In light of the reviews, which you will find at the end of this email, we would like to invite you to revise the work to thoroughly address the reviewers' reports.

As you will see, the reviewers wrote that the paper addresses an important question and provides novel and potentially significant findings. However, they each raised questions about some of the methodological choices and remaining sources of bias, as well as other points requiring clarification. In your revision, you should fully address the reviewers' comments through a combination of new analyses and textual changes.

Given the extent of revision needed, we cannot make a decision about publication until we have seen the revised manuscript and your response to the reviewers' comments. Your revised manuscript is likely to be sent for further evaluation by all or a subset of the reviewers.

In addition to these revisions, you will need to complete some formatting changes, which you will receive in a follow up email. A member of our team will be in touch with a set of requests shortly.

We expect to receive your revised manuscript within 3 months. Please email us (plosbiology@plos.org) if you have any questions or concerns, or would like to request an extension.

At this stage, your manuscript remains formally under active consideration at our journal; please notify us by email if you do not intend to submit a revision so that we may withdraw it.

**IMPORTANT - SUBMITTING YOUR REVISION**

Your revisions should address the specific points made by each reviewer. Please submit the following files along with your revised manuscript:

1. A 'Response to Reviewers' file - this should detail your responses to the editorial requests, present a point-by-point response to all of the reviewers' comments, and indicate the changes made to the manuscript.

*NOTE: In your point-by-point response to the reviewers, please provide the full context of each review. Do not selectively quote paragraphs or sentences to reply to. The entire set of reviewer comments should be present in full and each specific point should be responded to individually, point by point.

You should also cite any additional relevant literature that has been published since the original submission and mention any additional citations in your response.

2. In addition to a clean copy of the manuscript, please also upload a 'track-changes' version of your manuscript that specifies the edits made. This should be uploaded as a "Revised Article with Changes Highlighted" file type.

*Re-submission Checklist*

When you are ready to resubmit your revised manuscript, please refer to this re-submission checklist: https://plos.io/Biology_Checklist

To submit a revised version of your manuscript, please go to https://www.editorialmanager.com/pbiology/ and log in as an Author. Click the link labelled 'Submissions Needing Revision' where you will find your submission record.

Please make sure to read the following important policies and guidelines while preparing your revision:

*Published Peer Review*

Please note while forming your response, if your article is accepted, you may have the opportunity to make the peer review history publicly available. The record will include editor decision letters (with reviews) and your responses to reviewer comments. If eligible, we will contact you to opt in or out. Please see here for more details:

https://blogs.plos.org/plos/2019/05/plos-journals-now-open-for-published-peer-review/

*PLOS Data Policy*

Please note that as a condition of publication PLOS' data policy (http://journals.plos.org/plosbiology/s/data-availability) requires that you make available all data used to draw the conclusions arrived at in your manuscript. If you have not already done so, you must include any data used in your manuscript either in appropriate repositories, within the body of the manuscript, or as supporting information (N.B. this includes any numerical values that were used to generate graphs, histograms etc.). For an example see here: http://www.plosbiology.org/article/info%3Adoi%2F10.1371%2Fjournal.pbio.1001908#s5

*Blot and Gel Data Policy*

We require the original, uncropped and minimally adjusted images supporting all blot and gel results reported in an article's figures or Supporting Information files. We will require these files before a manuscript can be accepted so please prepare them now, if you have not already uploaded them. Please carefully read our guidelines for how to prepare and upload this data: https://journals.plos.org/plosbiology/s/figures#loc-blot-and-gel-reporting-requirements

*Protocols deposition*

To enhance the reproducibility of your results, we recommend that if applicable you deposit your laboratory protocols in protocols.io, where a protocol can be assigned its own identifier (DOI) such that it can be cited independently in the future. Additionally, PLOS ONE offers an option for publishing peer-reviewed Lab Protocol articles, which describe protocols hosted on protocols.io. Read more information on sharing protocols at https://plos.org/protocols?utm_medium=editorial-email&utm_source=authorletters&utm_campaign=protocols

Thank you again for your submission to our journal. We hope that our editorial process has been constructive thus far, and we welcome your feedback at any time. Please don't hesitate to contact us if you have any questions or comments.

Sincerely,

Taylor

Taylor Hart, PhD,

Associate Editor

PLOS Biology

thart@plos.org

------------------------------------

REVIEWS:

Reviewer #1: Summary

In their manuscript, "Human neuronal firing is modulated by the frequency of local field potential oscillations", the authors address an important question: how neural oscillations, decomposed into their two fundamental properties (frequency and phase) relate to single-neuron firing across brain regions. To approach this, they leverage a rich multimodal dataset containing both LFP and single-neuron recordings from five regions, offering a valuable opportunity to link region-level and cellular-level dynamics.

The authors report that a subset of neurons (15%) exhibited clear phase tuning, with spiking aligned to specific oscillatory phases. A detailed case example illustrates this phenomenon well, and further analyses confirm that the same neuron shows no preference for instantaneous frequency. Interestingly, ACC neurons demonstrate a higher prevalence of phase tuning. Beyond this, the authors also identify a complementary subset of neurons tuned to instantaneous frequency but not phase, and they classify three distinct types of frequency-related tuning. This dual approach provides a nuanced view of how oscillatory dynamics may guide spiking behavior. However, there're still several points that need to be considered

Major Points for Consideration

1.The manuscript convincingly develops the analysis of frequency tuning, while the treatment of phase effects could be expanded further. Since both phase and frequency are continuous variables, adopting a probability density framework for phase (similar to what was done for frequency) might provide a more balanced and informative comparison. This would be especially valuable because more neurons in the dataset exhibited phase tuning (54/357) compared to frequency tuning (27/357). Exploring phase tuning in greater detail could therefore yield deeper insight and make the study's conclusions even stronger.

Rather than contrasting spiking probability against a uniform phase distribution, the authors could present spiking probability density as a direct function of phase. This would clarify how probability mass is concentrated in preferred ranges and reveal whether spiking is sharply confined to narrow phase windows or more broadly distributed. Such an approach could significantly enrich the story of how phase modulates spiking.

2.Clarifying independence of phase and frequency tuning

The manuscript makes an important contribution by showing that phase tuning and frequency tuning are independent phenomena, likely reflecting distinct mechanisms. One challenge, however, is that instantaneous frequency and phase are not strongly correlated by definition: phase evolves continuously, while instantaneous frequency does not. This inherent difference may reduce the chance of detecting neurons tuned to both properties under the current framework.

It would strengthen the work to clarify how this methodological issue is accounted for, or to briefly discuss alternative approaches that might better capture potential overlap between frequency and phase tuning. Even acknowledging this limitation would help readers interpret the results more accurately.

3.Distribution of tuning across frequency ranges

The authors do an excellent job showing that frequency tuning is not simply a byproduct of spectral density. A similar level of care could be applied to one of their central claims: that 90% of phase- or frequency-tuned neurons occur at <10 Hz. This is an intriguing result, but it may be partly driven by bias if the LFP spends disproportionately more time in lower frequency ranges.

To strengthen this claim, the authors could quantify the proportion of time the LFP spends in each frequency band. Additionally, computing spike probability relative to the baseline prevalence of each frequency would test whether neurons fire disproportionately at certain frequencies, independent of how much time the signal spends there. This would help confirm whether the bias toward <10 Hz reflects genuine tuning or simply frequency distribution properties.

Minor Points

1.Including multiple axis views of the transparent brain in Figure 1A would make anatomical localization of recording sites clearer.

2.In Figure 1B, using a more intuitive color scheme (e.g., green for preferred phase ranges, grey otherwise) would highlight phase tuning more clearly. A similar approach could be applied to distinguish frequency-tuned from non-tuned neurons.

3.The "two-way KS test" would be better described as a "two-sample KS test."

This is a creative and well-designed study that makes an important contribution to understanding the relationship between oscillatory dynamics and neuronal spiking. The dataset is rich, the analyses are thoughtfully performed, and the results open new doors for linking cellular activity to population-level rhythms. With some additional analyses of phase effects and further clarification of frequency biases, the manuscript has strong potential to make a lasting impact in the field.

Reviewer #2: The authors investigate a fairly novel question using a very rare dataset: does the timing of human neurons align with the instantaneous frequency of the local field potential (LFP)? Given the relatively high variance in frequency that human LFPs exhibit, particularly in theta oscillations in the medial temporal lobe, this study is well posed to provide valuable information to researchers interested in temporally-mediated information encoding in the human brain. There are several issues that the authors should address, though, before the manuscript is ready for publication:

1.I am concerned be the overall predominance of very slow frequencies (1-2.5 Hz; Supplementary Figure 1), and implications this may have, since a large proportion of the small # of neurons showing any significant effect are within the 1-5 Hz range. I wonder if oscillations in this range are more likely to yield significant phase- and frequency- locking estimates due to the high autocorrelation in such slow signals. I don't think the circular shuffle fully addresses this possibility. Let us imagine a neuron whose firing rate ramps up slowly over the 5-minutes of recording time. At the same time, imagine that the LFP instantaneous frequency gradually decreases from 3 Hz to 2 Hz. Now, spikes will sample the low-frequency epochs more heavily, so the empirical spike-frequency histogram skews low. If you circularly shift the frequency trace, the ramp-up in firing will now align with higher or lower parts of the frequency trace arbitrarily, which will intrinsically result in lower spike-frequency coupling in the shuffled distribution than the empirical data. But that would not necessarily support the idea that spikes are "tuned" to low-frequencies, but just that the two variables systematically drifted in opposite directions slowly over time. I would bet this pattern is more likely in data that is predominantly very slow, as is the case in this dataset. The authors could attempt to shuffle the spike times themselves, using an ISI-based shuffling approach, or even show that the effects are correlated across time using a split-half analysis.

2.It is possible that I am misunderstanding how instantaneous frequency is extracted by ORCA, but it appears that very little information is provided about the amplitude of the signal at frequencies that are supposedly tuning spiking. The relationship of frequency tuning to the power spectrum should be explored (similar to Figure 6). Is there a relationship (or bias) between frequency estimates and power? I think a more convincing analysis would stratify frequency-tuning by instantaneous amplitude/power bins (e.g., low/medium/high) and test frequency tuning within each bin.

3.The authors show that phase-locked cells do not overlap with frequency-locked cells. But do phase-distributions systematically differ in recordings with high instantaneous frequency variance, in a way that might systematically prevent the identification of simultaneous phase- and frequency- locking?

4.I think it's interesting but somewhat hard to interpret that the authors only utilize the closest macro-electrode contact for their analyses. Most prior work uses the LFP recorded from the microwire (making it more truly "local"). How does this choice affect the authors results? How does the use of a common-average reference affect the results (compared to an arguably more local bipolar reference)?

5.Could "changes in instantaneous frequency" be explained by non-sinusoidal changes in waveform shape?

6.I am having a hard time parsing Supplementary Figure 6.

7.I recognize the difficulty in accounting for inter-subject differences (e.g. mixed-effects modeling) in a small dataset like this, but it would at least be helpful to know how many significant phase- and frequency- locking neurons are contributed by each individual subject.

8.The authors refer to frequencies below 10 Hz as "low theta", which is not in concordance with the literature.

9.Given the extremely rare dataset, authors should cite a highly relevant and recent work examining theta-phase locking in human neurons (PMID: 38948829).

Reviewer #3: The authors ask whether instantaneous frequency of intracranial EEG predicts activity of single units measures with micro-electrodes in human patients undergoing invasive epilepsy monitoring. The authors analyse data from 19 patients at rest, from which they analysed signald from 84 iEEG contacts and 357 units. Their Analyses show that both, frequency tunig and phase tuning of spikes to the intracranial EEG exists. Further, units are either frequency or phase tuned but not both. Both frequency and phase tuning occur in lower frequency bands <10 hz.

I think this paper contributes novel and important findings to the field and helps us to better understand the relationship between slower, local intracranial EEG fluctuations and spiking activity. Especially the finding on frequency but not phase-tuning in a subpopulation of units is novel.

I have no major concern but rather questions for clarifications mainly on methodological aspects. First, The authors use the ORCA as algorithm to extract instantaneous phase and frequency. As this method is central to obtain instantaneous estimates of dominant frequency and phase, I would appreciate a bit more detail on the motivation and benefits of this procedure, especially in light of the current paper. Do the authors think that more traditional (i.e, filter-hilbert in e.g. the theta range) spectral decomposition methods would result in different results and conclusions and why?

Another question relates to how local the low frequency oscillations are the authors record from the macro contacts. I think this is important state that the iEEG signal at the closest macro to the micro contacts is merely a proxy for the local field potential for neurons recorded with nearby microwires. One needs to bear in mind that the macro contact is still a few millimeters away from the microcontacts with which the authors record unit activity using BF-electrodes. A related question concerns the choice of reference for the macro-contact - did they authors re-reference the macro contact to second-closest macro on the same BF-electrode to isolate generators near the microwires?

Relatedly, the authors could have used lowpass filtered microwire recordings containing the actual local field potential. What was the reasoning for not using the microwire recordings, considering that there are accepted methods for spike removal, especially when estimating lower frequency oscillations in the LFP?

Minor things:

On page 15, the authors state they did not find any bursting neurons. This seems surprising. Do the authors have an explanation for that.

On page 16, bottom Canolty et al. 2012 is cited in text. I guess it should be in numbered format as well.

Line 466: The sentens starting with "Cohen deomonstrated lower-frequency…" is odd and might need rephrasing.

# Author comment: Author response to Decision Letter 2

**DOI:** 10.1371/journal.pbio.3003818.r003

23 Mar 2026

Attachment Submitted filename: Frequency_Tuning_ResponseLetter_final.pdf

# Peer review: Decision Letter 2

**DOI:** 10.1371/journal.pbio.3003818.r004

**Authors:** Taylor Hart, PhD

16 Apr 2026

Dear Dr Bartoli,

Thank you for your patience while we considered your revised manuscript "Human neuronal firing is modulated by the frequency of local field potential oscillations" for publication as a Research Article at PLOS Biology. This revised version of your manuscript has been evaluated by the PLOS Biology editors, the Academic Editor, and two of the original reviewers.

Based on the reviews, one of which was signed, we are likely to accept this manuscript for publication. Please also make sure to address the following data and other policy-related requests.

IMPORTANT: Please ensure that your next revision addresses the following points:

--------------------------

**Title:

Because the data in the study do not show direction of causality, we think it would be better to avoid the word 'modulates' in the title. Is the following modified version acceptable to you?

"Human neuronal firing reflects the frequency of local field potential oscillations"

**Financial disclosure statement:

Please confirm that all relevant grant numbers are included in the financial disclosure statement. Please also add links to the funding agencies.

**Ethics:

Please make the Ethics Statement the first subheading in the Materials and Methods section and include all ethical approval information here (these appear duplicated in the current version of the study). Please confirm that the study was conducted according to the principles expressed in the Declaration of Helsinki.

**Data:

Thank you for agreeing to include the processed data necessary to reproduce the figures and main findings. Please make this data available to us now and finalize the data statement (including the URL for publication).

At a minimum, we require that the processed, anonymized data related to the following figure panels be made available:

6CD

7ABC

Please also cite the location of the data clearly in all relevant main and supplementary Figure legends, e.g. “The data underlying this Figure can be found in S1 Data” or “The data underlying this Figure can be found in https://doi.org/10.5281/zenodo.XXXXX”

**Supplement:

Please upload the Supplementary Figures and Tables as separate Supporting Information files.

**Code availability:

Per journal policy, if you have generated any custom code or scripts during the course of this investigation, we require that you make it available without restrictions. Please ensure that the code is sufficiently well documented and reusable, and that your Data Statement in the Editorial Manager submission system accurately describes where your code can be found. Please also ensure that you choose a license for your code and include a Readme file.

--------------------------

As you address these items, please take this last chance to review your reference list to ensure that it is complete and correct. If you have cited papers that have been retracted, please include the rationale for doing so in the manuscript text, or remove these references and replace them with relevant current references. Any changes to the reference list should be mentioned in the cover letter that accompanies your revised manuscript.

In addition to these revisions, you may need to complete some formatting changes, which you will receive in a follow up email. A member of our team will be in touch with a set of requests shortly. If you do not receive a separate email within a few days, please assume that checks have been completed, and no additional changes are required.

We expect to receive your revised manuscript within two weeks.

To submit your revision, please go to https://www.editorialmanager.com/pbiology/ and log in as an Author. Click the link labelled 'Submissions Needing Revision' to find your submission record. Your revised submission must include the following:

- a cover letter that should detail your responses to any editorial requests, if applicable, and whether changes have been made to the reference list

- a Response to Reviewers file that provides a detailed response to the reviewers' comments (if applicable, if not applicable please do not delete your existing 'Response to Reviewers' file.)

- a track-changes file indicating any changes that you have made to the manuscript.

NOTE: If Supporting Information files are included with your article, note that these are not copyedited and will be published as they are submitted. Please ensure that these files are legible and of high quality (at least 300 dpi) in an easily accessible file format. For this reason, please be aware that any references listed in an SI file will not be indexed. For more information, see our Supporting Information guidelines:

https://journals.plos.org/plosbiology/s/supporting-information

*Published Peer Review History*

Please note that you may have the opportunity to make the peer review history publicly available. The record will include editor decision letters (with reviews) and your responses to reviewer comments. If eligible, we will contact you to opt in or out. Please see here for more details:

https://plos.org/published-peer-review-history/

*Press*

Should you, your institution's press office or the journal office choose to press release your paper, please ensure you have opted out of Early Article Posting on the submission form. We ask that you notify us as soon as possible if you or your institution is planning to press release the article.

*Protocols deposition*

To enhance the reproducibility of your results, we recommend that if applicable you deposit your laboratory protocols in protocols.io, where a protocol can be assigned its own identifier (DOI) such that it can be cited independently in the future. Additionally, PLOS ONE offers an option for publishing peer-reviewed Lab Protocol articles, which describe protocols hosted on protocols.io. Read more information on sharing protocols at https://plos.org/protocols?utm_medium=editorial-email&utm_source=authorletters&utm_campaign=protocols

Please do not hesitate to contact me should you have any questions.

Sincerely,

Taylor

Taylor Hart, PhD,

Associate Editor

thart@plos.org

PLOS Biology

REVIEWS

Reviewer #1: The authors have addressed all my questions satisfactorily. This will make a fine contribution.

-Rob Reinhart

Reviewer #2: The authors have addressed all my concerns and greatly clarified the rigor and specificity of these findings.

# Author comment: Author response to Decision Letter 3

**DOI:** 10.1371/journal.pbio.3003818.r005

30 Apr 2026

Attachment Submitted filename: Frequency_Tuning_ResponseLetter_final_auresp_3.pdf

# Editor report: Decision Letter 3

**DOI:** 10.1371/journal.pbio.3003818.r006

**Authors:** Taylor Hart, PhD

8 May 2026

Dear Dr Bartoli,

Thank you for the submission of your revised Research Article "Human neuronal firing varies with the frequency of local field potential oscillations" for publication in PLOS Biology. On behalf of my colleagues and the Academic Editor, Simon Hanslmayr, I am pleased to say that we can in principle accept your manuscript for publication, provided you address any remaining formatting and reporting issues. These will be detailed in an email you should receive within 2-3 business days from our colleagues in the journal operations team; no action is required from you until then. Please note that we will not be able to formally accept your manuscript and schedule it for publication until you have completed any requested changes.

Please note that, as we have commissioned a brief Primer article related to your study, we have declined early article posting on your behalf. This will allow us to coordinate publication of your study and the accompanying Primer.

Please take a minute to log into Editorial Manager at http://www.editorialmanager.com/pbiology/, click the "Update My Information" link at the top of the page, and update your user information to ensure an efficient production process.

PRESS

We frequently collaborate with press offices. If your institution or institutions have a press office, please notify them about your upcoming paper at this point, to enable them to help maximise its impact. If the press office is planning to promote your findings, we would be grateful if they could coordinate with biologypress@plos.org. If you have previously opted in to the early version process, we ask that you notify us immediately of any press plans so that we may opt out on your behalf.

We also ask that you take this opportunity to read our Embargo Policy regarding the discussion, promotion and media coverage of work that is yet to be published by PLOS. As your manuscript is not yet published, it is bound by the conditions of our Embargo Policy. Please be aware that this policy is in place both to ensure that any press coverage of your article is fully substantiated and to provide a direct link between such coverage and the published work. For full details of our Embargo Policy, please visit http://www.plos.org/about/media-inquiries/embargo-policy/.

Thank you again for choosing PLOS Biology for publication and supporting Open Access publishing. We look forward to publishing your study.

Sincerely,

Taylor

Taylor Hart, PhD,

Associate Editor

PLOS Biology

thart@plos.org
